# A Roadmap for the Human Oral and Craniofacial Cell Atlas

**DOI:** 10.1177/00220345221110768

**Published:** 2022-09-26

**Authors:** A.J. Caetano, Human Cell Atlas Oral and Craniofacial Bionetwork, A. Caetano, I. Sequeira, K.M. Byrd

**Affiliations:** 1 Centre for Craniofacial and Regenerative Biology, Faculty of Dentistry, Oral and Craniofacial Sciences, King’s College London, London, UK; 2 Institute of Dentistry, Barts Centre for Squamous Cancer, Barts and The London School of Medicine and Dentistry, Queen Mary University London, London, UK; 3 Lab of Oral and Craniofacial Innovation, Department of Innovation and Technology Research, ADA Science and Research Institute, Gaithersburg, MD, USA

**Keywords:** craniofacial, oral mucosa, palate, saliva, tongue, periodontium, bone, musculoskeletal, single cell genomics, multiomics, spatial biology, Human Cell Atlas

## Abstract

Oral and craniofacial tissues are uniquely adapted for continuous and intricate functioning, including breathing, feeding, and communication. To achieve these vital processes, this complex is supported by incredible tissue diversity, variously composed of epithelia, vessels, cartilage, bone, teeth, ligaments, and muscles, as well as mesenchymal, adipose, and peripheral nervous tissue. Recent single cell and spatial multiomics assays—specifically, genomics, epigenomics, transcriptomics, proteomics, and metabolomics—have annotated known and new cell types and cell states in human tissues and animal models, but these concepts remain limitedly explored in the human postnatal oral and craniofacial complex. Here, we highlight the collaborative and coordinated efforts of the newly established Oral and Craniofacial Bionetwork as part of the Human Cell Atlas, which aims to leverage single cell and spatial multiomics approaches to first understand the cellular and molecular makeup of human oral and craniofacial tissues in health and to then address common and rare diseases. These powerful assays have already revealed the cell types that support oral tissues, and they will unravel cell types and molecular networks utilized across development, maintenance, and aging as well as those affected in diseases of the craniofacial complex. This level of integration and cell annotation with partner laboratories across the globe will be critical for understanding how multiple variables, such as age, sex, race, and ancestry, influence these oral and craniofacial niches. Here, we 1) highlight these recent collaborative efforts to employ new single cell and spatial approaches to resolve our collective biology at a higher resolution in health and disease, 2) discuss the vision behind the Oral and Craniofacial Bionetwork, 3) outline the stakeholders who contribute to and will benefit from this network, and 4) outline directions for creating the first Human Oral and Craniofacial Cell Atlas.

## Introduction: The Human Cell Atlas Oral and Craniofacial Bionetwork

Oral and craniofacial tissues support the human face and thus the concept of our individual and collective identity; biologically, they coordinate essential functions to sustain life, including breathing, feeding, and communication. Though often preventable, oral diseases are increasingly burdensome worldwide, affecting over one-third of the globe with a disproportionate effect on socially disadvantaged populations. In addition, chronic oral inflammatory diseases, such as periodontal disease, have been increasingly associated with >60 systemic diseases, such as cardiovascular diseases, diabetes, cancer, pneumonia, inflammatory bowel diseases, obesity, and premature birth (Schifter et al. 2010; Beck et al. 2019; Byrd and Gulati 2021). To achieve the goal of improved and precise *whole-body health* will require the creative and dedicated application of new toolkits to understand the human body in health and disease states in combination with extensive in vivo animal studies and in vitro systems.

**Figure 1.**
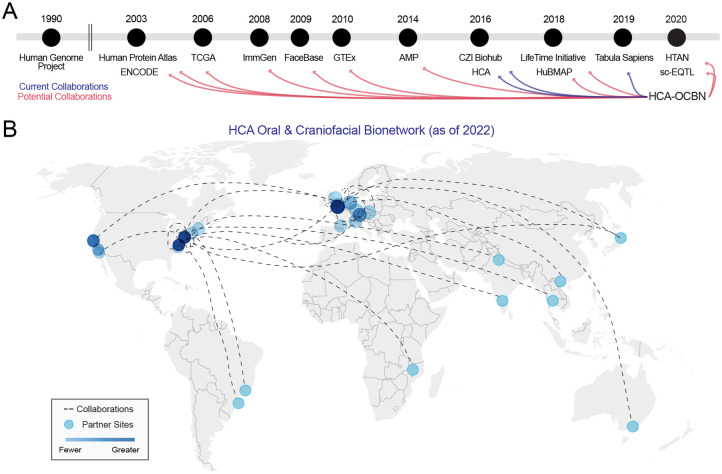
Collaboration opportunity across initiatives, networks, groups, and consortia. The Human Cell Atlas Oral and Craniofacial Bionetwork (HCA-OCBN; established 2020) is a collection of aligned investigators with the goal of mapping healthy human tissues of the oral and craniofacial complex. This network is using single cell and spatial multiomics to achieve these goals. (**A**) Since the Human Genome Project was completed in 2003, there have been several initiatives established that can benefit human oral and craniofacial research. There is enormous potential for fruitful collaboration between OCBN and initiatives such as FaceBase (established 2009), CZI Biohub (2016; Chan Zuckerberg Initiative), and HubMAP (2020; Human Biomolecular Atlas Program). Some of these groups are already collaborating with the OCBN (blue lines). (**B**) Collaboration within the OCBN can be as focused as sharing tissues and fluid samples or supporting computational analysis. As of May 2022, projects are growing monthly across new OCBN teams. Map generated from https://app.datawrapper.de/. AMP, Accelerating Medicines Partnership Program; ENCODE, Encyclopedia of DNA Elements; GTEx, Genotype-Tissue Expression project; HTAN, Human Tumor Atlas Network; ImmGen, Immunological Genome Project; sc-EQT, single-cell eQTLGen Consortium; TCGA, The Cancer Genome Atlas.

Recent advances in methods and the resolution of high-throughput single cell and spatial molecular profiling now appear to be the quantum leap that precision medicine has required, revolutionizing our ability to study tissue heterogeneity at a remarkable resolution and driving the scientific community to characterize all cells of the human body (Aldridge and Teichmann 2020). While the origins of the modern single cell revolution can be traced to quantitative polymerase chain reaction assays with individual neurons 3 decades ago (Eberwine et al. 1992), it was not until 2016 that the Human Cell Atlas (HCA; https://www.humancellatlas.org) initiative was officially launched (Fig. 1A). Since then, the HCA has established itself as an international and interdisciplinary collaborative effort to create reference cellular maps of the body across the life span (Regev et al. 2017; Regev et al. 2018).

The HCA consortium sits uniquely among a lineage of initiatives, networks, groups, and other multiomic-focused consortia that came after the Human Genome Project was completed in 2003 (Fig. 1A) as it does not direct its associated investigators’ studies. Despite this leadership structure, various HCA bionetworks and single cell and spatial biology consortia have made landmark scientific advances, including the discovery of new and rare human cell type annotations, referred to as “cell subtypes” (Montoro et al. 2018; Park et al. 2018; Aizarani et al. 2019; Litvinukova et al. 2020; McDonald et al. 2021), as well as disease-associated cell “states” (Kinchen et al. 2018; Ledergor et al. 2018; Sade-Feldman et al. 2018; Martin et al. 2019; Vieira Braga et al. 2019; Reynolds et al. 2021).

To support the investigation and inclusion of healthy oral and craniofacial tissues in the first draft of the HCA, the Oral and Craniofacial Bionetwork (OCBN) was founded in 2020 as a research network focused on postnatal adult oral and craniofacial tissues within the HCA (Fig. 1). Additionally, other craniofacial organs and hard tissues, such as the brain, eye, nasal cavity, face skin, adipose, and musculoskeletal system (Baldwin et al. 2021), are described in allied HCA bionetworks. Here, we 1) highlight recent collaborative efforts to employ new single cell and spatial approaches to resolve our collective biology at a higher resolution in health and disease, 2) discuss the vision behind the OCBN, 3) outline the stakeholders who contribute to and will benefit from this network, and 4) outline directions for creating the first Human Oral and Craniofacial Cell Atlas.

## A Roadmap for the Human Oral and Craniofacial Cell Atlas

### Defining Oral and Craniofacial Tissue Heterogeneity

The oral and craniofacial complex is supported by highly diverse tissue niches, composed of epithelia, blood and lymphatic vessels, cartilage, bone, ligaments, and muscles, as well as adipose and peripheral nervous tissue (Nanci 2018). This anatomy has been well described for decades (Nanci 2018); however, recent genetic and genomics approaches based on mouse models have found intra- and interspecific niche heterogeneity among the periodontium (Nagata et al. 2021; Zhao and Sharpe 2021), tooth (Sharir et al. 2019; Takahashi et al. 2019; Chiba et al. 2020; Krivanek et al. 2020), salivary glands (Song et al. 2018; Hauser et al. 2020; Sekiguchi et al. 2020), palate (Byrd et al. 2019; Li et al. 2019; Han et al. 2021), buccal mucosa (Jones et al. 2019), and tongue (Tabula Muris et al. 2018; Almanzar et al. 2020). Some of these data have recently been integrated (Huang et al. 2020; Williams et al. 2021), though focused on healthy barrier epithelia, and there appears to be common and unique cell types among these niches.

**Figure 2.**
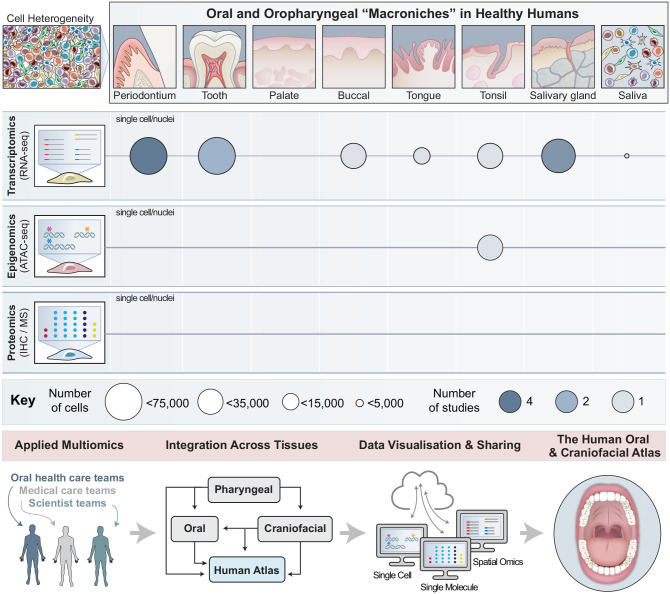
A blueprint for the Human Oral and Craniofacial Cell Atlas. Oral and craniofacial tissue niches are incredibly diverse, including periodontium, tooth, palate, buccal mucosa, tongue, tonsils, salivary glands, and fluid from the saliva—these do not account for microniches that are unaccounted for among these tissue spaces. Each niche is supported by some combination of epithelia, cartilage, bone, ligaments, muscles, adipose tissue, blood and lymphatic vessels, and nerves, and these tissues are harmoniously integrated into the vital functions of communication, feeding, breathing, defense, sensing, and early digestion. The Human Oral and Craniofacial Cell Atlas, supported by the Oral and Craniofacial Bionetwork (OCBN), aims to create comprehensive and integrated cell atlases to understand the common and unique cell types that support these niches in health and to uncover which cell types and networks are affected in disease. We will do this using single cell and spatial multiomic approaches (transcriptome, epigenomic, proteomic), and we will incorporate additional omics technologies as assays are refined and available. Thus far, most work from the OCBN and others has focused on single cell transcriptomic (scRNAseq) and epigenomic (ATACseq) approaches from healthy adults, including nearly all major tissue niches and saliva. Work is currently being done with the OCBN for collecting, integrating, visualizing, sharing, and applying the knowledge gained from these early studies. The number of studies developed so far and the total number of cells profiled are depicted under each tissue. Tissue illustrations inspired by Huang et al. (2021). Credit: Heather McDonald, BioSerendipity, LLC.

**Figure 3.**
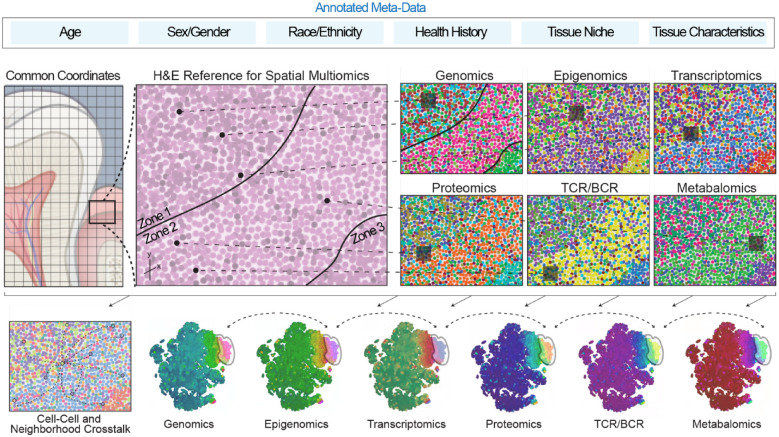
Applied spatial multiomics for coordinated and integrated analyses. Coordinated efforts across the Human Cell Atlas are working toward building a consensus of the necessary metadata (clinical and biological) to generate a common coordinate framework for the human body. Future work including these additional layers of data will highlight the diversity of cell types and states across humans considering age, sex, race, ethnicity, ancestry, and oral and systemic health history, as well as the specific niche, tissue orientation, and health status of that niche (healthy, inflamed) at the time of sample collection. There is an immense need to interrogate a higher number of molecular dimensions or human tissues—genome, transcriptome, epigenetic modifications, proteome, T cell receptor and B cell receptor repertoires (TCR/BCR), and metabolome of the collected sample itself—as no single “omics” technology can fully define the complexity of molecular mechanisms, but taken together, these integrated data have the potential to provide a more comprehensive landscape of basic biological processes and human disease. Multimodal sequencing has the capacity to move the field from descriptive “snapshots” toward a mechanistic understanding of gene regulatory networks and, importantly, to refine sources of cellular heterogeneity as already applied to the immune system. The use of multimodal single cell and spatial multiomics is therefore revolutionizing our understanding of cellular biology; however, relying on the dissociation of cells from their natural tissue environment limits our ability to understand the role of intrinsic and extrinsic factors that underpin cellular communication and organ function. Spatial multiomic approaches, which include information on the location of cells, will still need to be integrated with these single cell multiomic maps.

Importantly, oral and craniofacial tissue heterogeneity is not defined at just the level of biology (i.e., molecular, cellular, tissue, organ) as it is well established that human sex, age, and ancestry influence tissue physiology. There is an urgent need to increase representation in scientific research, as ~85% of today’s genomic data are derived from European ancestry; therefore, it is crucial to account not only for tissue heterogeneity but also for expanding into human geographic, sex/gender, age, ethnic, and ancestral diversity in the donor data sets (Figs. 2, 3). The OCBN takes this mission seriously among its investigators and study participants (Fig. 1B) with one funded study focused on generating healthy oral and craniofacial multiomic reference data from at least 8 global ancestries.

### Collecting Oral and Craniofacial Atlases as Reference Data Sets

Significant progress has been made by the HCA community with the current total of profiled cells to date numbering about 40 million cells from 15 major organs (Lindeboom et al. 2021) (https://data.humancellatlas.org/). While there are an estimated 40 trillion cells in the human body, we estimate that there are about 2 trillion in the oral and craniofacial tissues. Although progress in the single cell profiling of human oral tissues has been slow in comparison, researchers have made significant progress as of 2022, publishing atlases of the oral mucosa, including the buccal mucosa, tongue, and gingiva (Caetano et al. 2021; Huang et al. 2021; Williams et al. 2021; Tabula Sapiens et al. 2022); major and minor salivary glands (Huang et al. 2021; Chen et al. 2022; Costa-da-Silva et al. 2022; Tabula Sapiens et al. 2022); dental pulp (Krivanek et al. 2020; Pagella et al. 2021; Opasawatchai et al. 2022); periodontal ligament (Pagella et al. 2021); tonsil (King et al. 2021); and even saliva itself (Choudhury et al. 2020; Fig. 2). Additional craniofacial niches, such as the temporomandibular joint and skeletal muscle, are planned and being executed as well.

In sum, these and other active studies have already identified regional differences in cellular types, proportions, states, phenotypes, and niche-dependent interactions and highlighted the importance of the tissue extracellular microenvironment in shaping the identity of resident epithelial, stromal, and immune cells. Within 18 mo (between September 2020 and March 2022), OCBN made significant strides forward to the modern age of single cell biology (Aldridge and Teichmann 2020), including nearly >250,000 cells representing the first cell types from 8 distinct niches from healthy adults (Fig. 2, Table). Within the OCBN, multidisciplinary teams are already conducting experiments incorporating single cell and spatial multiomics in pediatric and adult human subjects. Importantly, the rapid development of spatial technologies, such as sequencing- and FISH-based technologies, is now permitting the use of fresh-frozen and fixed paraffin-embedded samples, opening the possibility for analyzing human tissues from archived biobanks (Fig. 3).

**Table.** Human Oral and Craniofacial Single Cell and Spatial Multiomic Data Sets (2020–2022).

Study	Sample Type	Anatomic Information	Method	Clinical Annotations	Cell No.	Donors	Cell Ontology Class	Public Repository
Huang et al. (2021)	Gingival mucosa	Upper left maxillary lingual interproximal papilla	10x scRNA-seq	Gingivitis	6,683	4	Basal keratinocyte 1 / 2 / 3 / 4Basal cycling keratinocyteSuprabasal keratinocyteMelanocyte	https://www.covid19cellatlas.org/byrd20/
							Merkel cell	
							Langerhans cell	
							Arterial endothelium	
							Capillary endothelium	
							Fibroblast	
							Lymphatic endothelium	
							Smooth muscle cell	
							Macrophage	
							Dendritic cell 1 / 2	
							Activated dendritic cell	
							Plasmacytoid dendritic cell	
							Mast cell	
							Mucosal-associated invariant T cell	
							Natural killer cell	
							Cytotoxic T cell 1	
							Helper T cell	
							Regulatory T cell	
							γδ T cell	
							T/NK cycling cell	
							B cell	
Caetano et al. (2021)	Gingival mucosa	Upper right maxillary buccal margin	10x scRNA-seq	Healthy, periodontitis	12,411	4	Epithelial cell 1 / 2Basal cycling keratinocyte	GSE152042
							Fibroblast 1 / stromal (S0)	
							Fibroblast 2 / stromal (S1)	
							Fibroblast 3 / stromal (S2)	
							Fibroblast 4 / stromal (S4)	
							Fibroblast 5 / stromal (S6)	
							Myofibroblasts	
							Endothelial cell 1 / 2	
							Perivascular cell	
							Pericytes	
							Macrophage 1 / 2	
							Dendritic cell	
							Plasmacytoid dendritic cell	
							Mast cells	
							T cells	
							IgG B cells	
							Follicular B cell	
							Memory B cell	
Williams et al. (2021)	Gingival mucosa	Upper right/left buccal margin, lower right buccal margin	10x scRNA-seq	Healthy, periodontitis	88,140	21	Epithelial cell 1 / 2 / 3MelanocyteFibroblast 1 / 2 / 3 / 4 / 5	https://oral.cellatlas.ioGSE164241
							Smooth muscle cell	
							Vasculature endothelium cell 1 / 2 / 3 / 4	
							Lymphatic endothelium	
							αβ CD4+ T cell	
							Helper T cell 17	
							Mucosal-associated invariant T cell	
							αβ CD8+ T cell	
							γδ T cell	
							Regulatory T cell	
							Natural killer cell	
							Neutrophil	
							Macrophage	
							Migratory dendritic cell	
							Mast cell	
							Plasmacytoid dendritic cell	
							B cell	
							Plasma cell	
Williams et al. (2021)	Buccal mucosa	Left buccal mucosa	10x scRNA-seq	Healthy	34,999	8	Epithelial cell 1 / 2 / 3Melanocyte	https://oral.cellatlas.ioGSE164241
							Fibroblast 1 / 2 / 3 / 4	
							Smooth muscle cell	
							αβ CD4+ T cell	
							Helper T cell 17	
							Mucosal-associated invariant T cell	
							αβ CD8+ T cell	
							γδ T cell	
							Regulatory T cell	
							Natural killer cell	
							Neutrophil	
							Macrophage	
							Migratory dendritic cell	
							Mast cell	
							Plasmacytoid dendritic cell	
							B cell	
							Plasma cell	
Tabula Sapiens et al. 2022	Major salivary glands	Parotid; submandibular	10x scRNA-seq; Smartseq2	Healthy	27,199	2	Basal keratinocyte cellAcinar cell	https://tabula-sapiens-portal.ds.czbiohub.org
							Salivary gland cell	
							Duct epithelial cell	
							Ionocyte	
							Myoepithelial cell	
							Fibroblast	
							Endothelial cell	
							Lymphatic endothelial cell	
							Pericyte	
							Adventitial cell	
							Macrophage	
							Monocyte	
							Neutrophil	
							NK cell	
							B cell	
							Naive B cell	
							Plasma cell	
							Memory B cell	
							T cell	
							CD4+ helper T cell	
							αβ CD4+ T cell	
							αβ CD8+ T cell	
Huang et al. (2021)	Minor salivary glands	Labial minor	10x scRNA-seq	Healthy individuals	7,107	5	Basal keratinocyteMucous acinar cell	https://www.covid19cellatlas.org/byrd20/
							Serous acinar cell	
							Duct epithelial cell	
							Ionocyte	
							Myoepithelial cell	
							Fibroblast	
							Arterial endothelium	
							Capillary endothelium	
							Venule endothelium	
							Pericyte	
							Smooth muscle cell	
							Glial cell	
							Macrophage 1 / 2	
							Mast cell	
							Cytotoxic T cell 1 / 2	
							Helper T cell 1	
							Dendritic cell 2	
							B cell	
							Plasma cell	
							Erythrocyte	
Costa-da-Silva et al. (2022)	Minor salivary glands	Labial minor	10x scRNA-seq	Healthy individuals	21,402	4	Serous acinar cell	GSE180544
							Seromucous acinar cell	
							Mucous acinar cell	
							Ductal epithelial cell	
							Fibroblast	
							Myoepithelial cell	
							Pericyte/myofibroblast	
							Vasculature endothelium	
							Lymphatic endothelium	
							Smooth muscle cell	
							Myeloid immune cell	
							Plasma cell	
							T cell	
Pagella et al. (2021)	Periodontium	Periodontal ligament third molars	10x scRNA-seq	Healthy individuals	2,883	5	Epithelial cell 1 / 2 / 3 / 4 / 5Mesenchymal stem cell	https://github.com/TheMoorLab/Tooth
							Fibroblast	GSE161267
							Endothelial cell 1 / 2	
							Schwann cell	
							Immune cell 1 / 2 / 3	
							Erythrocyte	
King et al. (2021)	Tonsil	Pediatric tonsils	scRNA-seq; scVDJ	Recurrent tonsilitis; obstructive sleep apnea	32,607	7	CD4+ NCM T cellCD4+ T cellTfH, T cell	https://www.tonsilimmune.orgE-MTAB-8999E-MTAB-9003
							TfR T cell	E-MTAB-9005
							Regulatory T cell	
							CD8+ NCM T cell	
							CD8+ cytotoxic T cell	
							TIM3+ DN T cell	
							Innate lymphoid cell	
							NK Cell	
							T cell cycling	
							Macrophage precursor cell	
							Macrophage 1 / 2 / 3	
							Dendritic cell 1	
							Plasmacytoid dendritic cell	
							Follicular dendritic cell	
							FCRL4+ marginal B cell	
							Activated B cell	
							Marginal B cell	
							Naïve B cell	
							PreGC B cell	
							FCRL3-high B cell	
							GC B cell	
							DZ GC B cell	
							B cell cycling	
							Pre-plasmablast	
							LZ GC B cell	
							Plasmablast	
Choudhury et al. (2020)	Saliva	Adult saliva	Smart-seq2	Healthy individuals	~2,000	3	Neutrophil	https://data.humancellatlas.org/explore/projects/60ea42e1-af49-42f5-8164-d641fdb696bc
Tabula Sapiens et al. 2022	Tongue	Anterior, posterior	10x scRNA-seq; Smartseq2	Healthy individuals	15,020	3	Basal keratinocyte cellEpithelial cell	https://tabula-sapiens-portal.ds.czbiohub.org
							Keratinocyte	
							Fibroblast	
							Arterial endothelial cell	
							Capillary endothelial cell	
							Venule endothelial cell	
							Lymphatic endothelial cell	
							Pericyte	
							Skeletal muscle cell	
							Schwann cell	
							Immune cell	
Krivanek et al. 2020	Dental pulp, apical papilla	Third molars	10x scRNA-seq; Smartseq2	Healthy individuals	41,673	7	Odontoblast	http://pklab.med.harvard.edu/ruslan/dental.atlas.html
							Peri-odontoblastic cell	GSE146123
							Periodontal ligament cell	
							Endothelial cell	
							Perivascular cell	
							Pulpal cell	
							Glial cell	
							Macrophage	
							Neutrophil	
							NK cell	
							Lymphocyte	
							Cycling cell	
Pagella et al. (2021)	Dental pulp	Third molars	10x scRNA-seq	Healthy individuals	32,378	5	Epithelial cell	https://github.com/TheMoorLab/Tooth
							Mesenchymal stem cell 1 / 2 / 3	GSE161267
							Fibroblast 1 / 2 / 3 / 4 / 5 / 6	
							Endothelial cell 1 / 2 / 3 / 4 / 5	
							Odontoblast cell	
							Non-myelinating Schwann cell	
							Myelinating Schwann cell	
							Immune cell 1 / 2 / 3 / 4 / 5	
							Erythrocyte	
Opasawatchai et al. (2022)	Dental pulp	Third molars	10x scRNA-seq	Healthy and carious	6,810	4	Naive CD4/CD8 T cellEarly odontoblastMacrophage	GSE185222https://github.com/vclabsysbio/scRNAseq_Dentalpulp
							Odontoblast	
							CD4 T cell	
							CD8 T cell	
							Naive B cell	
							Erythrocyte	
							NK cell	
							Granulocyte	
							HSCs	
							Vascular endothelium	
							CD16 mono	
							Immature erythrocyte	
							Plasmacytoid dendritic cell	
							Plasma cell	
Chen et al. (2022)	Major salivary glands	Parotid	10x scRNA-seq	Healthy	16,052	1	B cellT cellFibroblast	GSE188478https://github.com/miao-OvO/PG-scRNA-seq
							NK cell	
							Serous acinar cell	
							Myeloid cell	
							Plasma cell	
							Ductal epithelial cell	
							Vascular endothelium	
							Myoepithelial cell	

Currently available human data sets contributed to public repositories, including the Human Cell Atlas Data Coordination Portal (Choudhury et al. 2020; Krivanek et al. 2020; Caetano et al. 2021; Huang et al. 2021; King et al. 2021; Pagella et al. 2021; Williams et al. 2021; Chen et al. 2022; Costa-da-Silva et al. 2022; Opasawatchai et al. 2022; Tabula Sapiens et al. 2022).

While early efforts have primarily focused on the adult oral soft tissues, craniofacial structures such as the cranial ganglia, cranial vault, cranial base, nasopharynx, nasal bones and cartilages, neck, pinna, and middle ear bones will be essential to allow a more detailed investigation into the molecular mechanisms controlling tendogenesis, chondrogenesis, and osteogenesis, which support craniofacial function and enhance cranial skeletal repair, thereby leading to a better understanding of craniofacial anomalies. Achieving these goals will require more collaborative efforts among established and new OCBN investigators, other HCA networks, as well as other consortia.

### Illuminating Intercellular Communication Networks between Defined Cell Types

With this reference data set, we aspire to accelerate discoveries in the domains of basic and clinically applied research with further downstream analyses. Expression profiling of different cell types in adult human tissues has shown how intercellular communication contributes to tissue function by coordinating cell functions in development and homeostasis; thus, when there are signaling defects, disease will follow. The study of intercellular communication has significantly accelerated with advances in the single cell field with several studies discovering novel signaling, mediating cellular differentiation and immune responses. Intercellular crosstalk has been investigated in oral tissues, with OCBN studies demonstrating how in periodontal disease there is a shift in the transcriptional signatures of stromal and epithelial oral mucosa cells to an inflammatory profile (Caetano et al. 2021; Williams et al. 2021). In disease, endothelial cells also showed upregulation of pathways related to lymphocyte adhesion and chemokine signaling (Williams et al. 2021).

Given that most cellular crosstalk is spatially restricted with signals working from 0 to 200 µm, spatial transcriptomics data are essential to understand intercellular communication in healthy and diseased tissues. To investigate how surrounding cells may regulate signaling, several computational methods have been recently developed to integrate spatial information with ligand-receptor analyses (Efremova et al. 2020; Dries et al. 2021). However, translating this to the clinic will require harmonized and consistent cell type annotation among different human atlases to allow for accurate intercellular communication network modeling, as disease networks consistently are sequenced and added to the HCA data sets. Harmonized nomenclature and annotation of cell types/states when integrating different source organ atlases is one of the current efforts of the HCA to achieve a unified reference cell ontology across tissues (Osumi-Sutherland et al. 2021).

### Annotated Clinical and Biological Metadata for a Common Coordinate Framework

While these initial OCBN projects have started to construct high-resolution maps of organs and tissues, there is an unmet need to integrate these atlases to interrogate analogous cellular components across tissue niches and to develop a common coordinate framework for the healthy oral and craniofacial human tissues (Fig. 3). So far, most annotations of OCBN single cell genomics data sets have distinct cell type annotations, even within the same tissue (Table). This discordance makes it difficult to relate findings among studies, highlighting the need for a common “language” for cell annotation. This framework will aim to address clinical and spatial variability within studies and allow for more accurate and precise comparisons among data sets across the human body. Relevant clinical metadata will include but not be limited to donor gender, sex, ethnicity, age, tissue type, relevant clinical data, and technology used; spatial data will define the position, tissue plane, site, and size of anatomic structures (Table).

Furthermore, future work incorporating these clinical and biological data will highlight the niche-specific cellular diversity that may help to explain why some oral diseases manifest in some oral and craniofacial niches while sparing others. For example, understanding niche-specific cellular heterogeneity in pediatric tissues from neonatal to infancy, juvenile, and adolescence periods will allow the identification of cell states and cell lineages involved in tissue maturation and a better understanding of early disease onset in childhood. However, given the vast literature on immune training in early development, these efforts should also reveal the mechanisms of healthy and pathologic aging that may lead to early and accurate prognostic tools allowing for early intervention, similar to what is proposed by the LifeTime Initiative (Rajewsky et al. 2020, 2021).

This additional level of annotation is already relevant. For example, the concept of structural immunity (i.e., niche specific) has recently been described across the body, suggesting that each tissue’s cell-specific composition can instruct its niche-distinct immune response (Krausgruber et al. 2020). This lens will provide a new framework for interrogating human disease by mapping disease risk genes and for predicting cell type–specific and coregulated gene modules, as recently described in periodontitis (Williams et al. 2021). For the long-term success of the OCBN, high-quality and widely available single cell and spatial multiomic data sets will need to be published with detailed metadata for each experiment and study.

### Integrated Analyses within and across Multimodal Data

A fundamental challenge when constructing biological systems is the correct definition of cell state, which is achieved by applying complementary approaches, such as molecular characterization (transcripts, distribution of chromatin marks and proteins) and functional testing (Fig. 3). Understanding cell-specific activity further requires proteomics, metabolomics, and functional assays to provide a direct readout of cellular activity. Furthermore, there is a need to interrogate a higher number of molecular dimensions. No single “omics” technology can fully define the complexity of molecular mechanisms, but taken together, these integrated data have the potential to provide a more comprehensive landscape of basic biological processes and human disease. Multimodal measurements, where distinct molecular parameters can be interrogated in the same cell, have been recently developed and are now allowing us to characterize cells, cell states, and transitions between cell states across multiple levels of regulation.

Multimodal sequencing has the capacity to move the field from descriptive “snapshots” toward a mechanistic understanding of gene regulatory networks and, importantly, to refine sources of cellular heterogeneity as already applied to the immune system (Hao et al. 2021). The use of multimodal single cell omics is therefore revolutionizing our understanding of cellular biology; however, relying on the dissociation of cells from their natural tissue environment limits our ability to understand the role of intrinsic and extrinsic factors that underpin cellular communication and organ function. Indeed, today in clinical settings, histopathology is a standard diagnostic tool as many diseases are defined by abnormal cellular organization. Additionally, many scientific discoveries rise from the understanding that cellular organization in tissues is highly connected to biological function. Thus, combining single cell molecular measurements with histology and microscopy assays will be required to ultimately generate biological insights into human health and disease.

Overcoming data set–specific batch effects through data integration of such population-level single cell data has remained a limitation. New computational tools recently developed outside the OCBN address this (Sikkema et al. 2022), allowing for the integration of multimodal health and disease data sets. However, computational tools to integrate high-resolution molecular and spatial information are still being established; there is no clear method that accounts for anatomic differences among individuals. The advantage of these newer integration assays is for the annotation of regional/cell molecular identities within the tissue architecture, unravelling cell-cell communication with a spatial context and clarifying tissue microniches, now referenced as “cell neighborhoods” within tissues (Nitzan et al. 2019).

Understanding the cellular context, including extracellular components and signaling molecules that contribute to organ homeostasis, will help to further identify the functions of specific cell types and interactions as well as provide mechanistic insights into fundamental biological processes in health and disease. Next, it will be necessary to integrate human multiomics data with common model organisms as well as patient-derived experimental disease models during the progression from health to disease. The interrogation of these in parallel will be essential for functional assays for data validation and the development of new testable hypotheses, ultimately accelerating targeted follow-up studies to enter the clinical research space.

### Clinically Relevant Innovation, Discovery, and Collaboration

For the construction of future oral and craniofacial atlases in disease, it remains paramount to assess the functions, gene expression, and intercellular interactions of all resident cells in healthy tissue as a reference. This level of annotation will allow for a clearer understanding of how these processes are disrupted in disease states. For instance, small subsets of cells are important in the pathogenesis of a variety of complex diseases (Regev et al. 2017), and studying the breakdown of immune mechanisms and dysregulated proinflammatory pathways on a cell-by-cell basis presents an opportunity to understand how perturbed molecular pathways and processes can lead to disease. Understanding these processes may identify molecular mechanisms that lead to improved targetedtherapies—for instance, in human craniofacial birth defects such as orofacial clefting or craniosynostosis. This is foundational knowledge that we intend to be publicly available to advance oral health across the globe.

The clinical significance of single cell approaches has been successfully demonstrated in various human diseases by allowing the identification of disease-associated cell phenotypes—for example, malignant tumor cells within a tumor’s mass (Tirosh et al. 2016) or the identification of immune cells that can predict clinical outcomes and enhance treatment strategies (Sade-Feldman et al. 2018). In the oral and craniofacial region, there are now glimpses of what is possible. For example, stromal cells promoting neutrophil migration in health that expand in disease have been identified (Williams et al. 2021), and an IgG plasma B cell response was identified as a hallmark of periodontitis (Caetano et al. 2021; Williams et al. 2021). The impact of single cell approaches in understanding human oral disease was further demonstrated during the COVID-19 pandemic, when the oral cavity was proved to be an important site for infection with saliva as a potential route of transmission (Huang et al. 2021). This is a minute sampling of the ever-growing clinical advances made possible through single cell approaches.

Many oral conditions will benefit from the molecular characterization of cellular subpopulations. It will provide valuable insights into factors that affect disease progression of head and neck tumors, such as oral carcinoma; potentially malignant disorders of the oral cavity, such as proliferative verrucous leukoplakia (Thompson et al. 2021); oral mucosal diseases, such as lichen planus and vesiculobullous diseases; salivary gland disorders, such as Sjögren’s syndrome; odontogenic and bony pathologies; and trisomy 21. Deepening our understanding of molecular characterization of cellular subpopulations will uncover the processes involved in oral manifestations of systemic disease, especially gastroenterology diseases such as Crohn’s disease, rheumatologic conditions such as lupus and Sjögren’s disease, and systemic hematologic diseases including white and red cell dyscrasias, sickle cell anaemia, or leukemia. To succeed in achieving these aims, the network actively seeks to engage all stakeholders, including patients, oral physicians, researchers, cell biologists, and data scientists, all of whom share in the collective vision of developing our understanding of oral health and disease—all while adhering to our values of transparency and open science (Regev et al. 2018).

## Discussion

### A Phased Strategy for the OCBN

Organizing such a large-scale project is dependent on a multidisciplinary approach relying on the close collaboration between oral surgeons and oral health care providers (to devise quality metrics to obtain and collect clinical samples), with wet laboratory scientists and bioinformaticians (for sample processing, data processing, and analysis). From the clinician’s view, it is crucial to carefully consider 1) sample collection criteria checkpoints, such as medical history screening; 2) the recording of the precise anatomic location of each sample; and 3) review and collection of associated donor and sample metadata, including health and disease states. Furthermore, this sort of collaboration will accelerate a shift from description to knowledge and the solving of complex clinical questions.

While work from the OCBN has just begun to reveal the diverse tissues and fluids of the oral and craniofacial complex (phase 1), we are already planning to conduct studies that illuminate how these niches harmoniously integrate into the vital functions of communication, defense, breathing, and digestion (phase 2). The OCBN is committed to establishing an initial version of the oral and craniofacial atlas within 2 y with phase 1 of all other HCA bionetwork atlases. As such, the OCBN is currently integrating the existing OCBN data sets (Table) with new unpublished data using harmonized nomenclature and annotation of cell types.

By taking an agnostic approach to tissue and organ physical location (e.g., not oral but airway; not skin but stratified squamous epithelia) should allow for shared discoveries to accelerate human health clinical benefit. For example, while the oral cavity is an important ecosystem, it is also a crossroads that is affected by the condition of other, even distant, body sites. This is not a surprise; these tissues are intimately connected to the nervous, immune, cardiovascular, and endocrine systems (Tirosh et al. 2016). In a bidirectional manner, the condition of the oral cavity can affect distant sites as well (Beck et al. 2019). Phase 1 (oral and craniofacial atlas) and phase 2 (integration of oral and other tissue atlases) of the OCBN will complement other consortia and other model organism databases, such as FaceBase (Samuels et al. 2020). Furthermore, although the OCBN occupies a unique gap within current multiomic initiatives (Fig. 1A), partnership with these other groups outside the HCA, within the HCA, and within the OCBN (Fig. 1B) is essential to the successful integration of the healthy oral and craniofacial data and will facilitate clinical applications.

**Figure 4.**
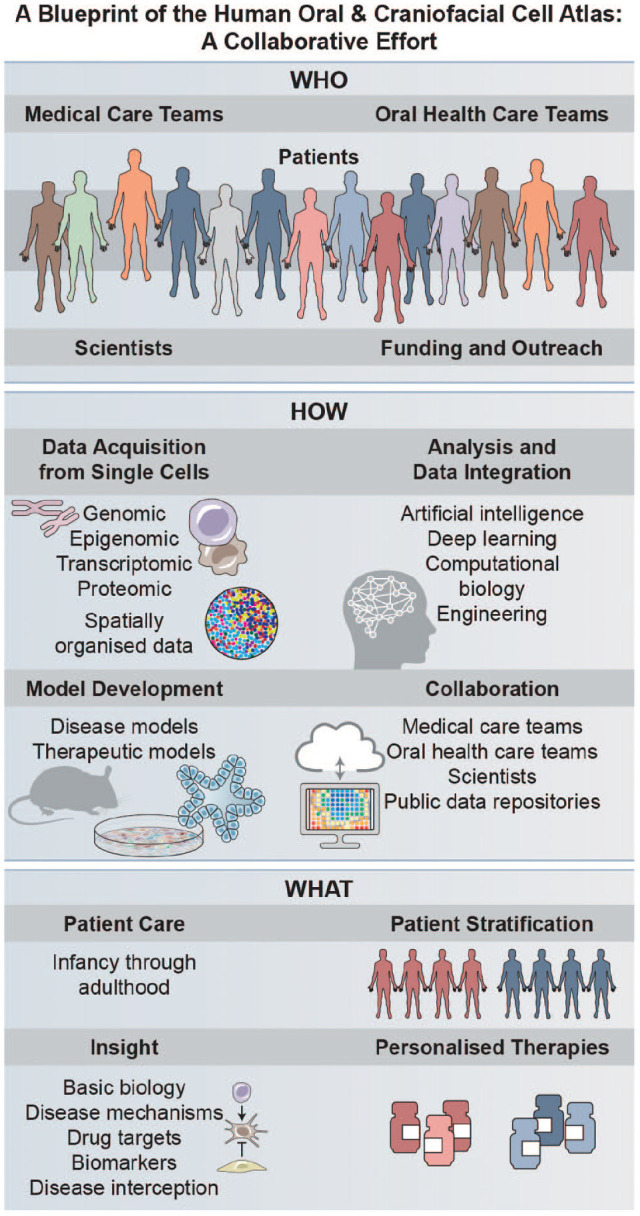
The vision for a globally representative Human Oral and Craniofacial Cell Atlas. To accomplish the goals of the OCBN, we will require the development of inter- and intrainstitute partnerships to connect oral health care teams (namely but not exclusively: oral pathology, oral surgeons and periodontists, endodontics, oral medicine, oral radiology, and general dentists) with medical care teams (namely but not exclusively: ear/nose/throat, dermatology, gastroenterology, pulmonology, allergists, endocrinologists, pediatricians, neurologists, pathologists, clinical genetics, oncologists, radiologists as well as internal and emergency medicine providers). This collaborative approach will allow us to lead the burgeoning field of integrated precision oral health through the application of innovative investigative approaches (single cell, spatially mapped genomics, epigenomics, transcriptomics, and proteomics as well as other “omics” approaches such as metabolomics and microbiomics). Furthermore, defining oral and craniofacial tissue mucosal niches across the life span and building on those reference data will provide unparalleled opportunities to interrogate the mechanisms of common and rare oral and craniofacial diseases across the life span. We will be working collaboratively with other research centers, investigator networks, and related consortia to make data and samples available to the broader research community to address common and rare health concerns globally; to develop scalable representative disease and therapeutic models; and to further integrate, analyze, and discover actionable targets through artificial intelligence/machine learning technologies. In total, our network should have a positive impact on precision medicine for all patients. Credit: Heather McDonald, BioSerendipity, LLC.

Progress toward comprehensive mapping of oral and craniofacial tissues requires not only careful experimental design to robustly capture variation within and across individuals but, importantly, a physiologic insight to interpret data and curate data sets. To this end, it is essential to increase dialogue among biologists, computational data scientists, clinicians, pathologists, and statisticians to achieve a consensus on data curation and cell annotation and to deliver data analysis platforms that are relevant and user-friendly (Fig. 4). Moreover, public engagement involving different communities and research participants will be essential to articulate the motivations of the project and to raise awareness of its ambitions and research priorities. In addition, public data portals and biorepositories that enable users to easily access and analyze HCA data are essential to the HCA goal of inclusivity, integrity, and data sharing (https://data.humancellatlas.org; https://oral.cellatlas.io; https://www.covid19cellatlas.org/byrd20/).

Finally, the Human Oral and Craniofacial Cell Atlas encourages and supports the participation of scientists and clinicians from countries around the globe by recognizing the need to integrate different ethnicities, environments, and regional diseases, and we invite any interested stakeholder to join the network, participate in our meetings, and contribute data for the integrated atlas (see contact details in Conclusions). We are aware of potential challenges and limitations, including available resources and tissue sampling, but we are committed to shared protocols (Greenwell-Wild et al. 2021), open and immediate data release, and prioritizing international collaborative work. Collective development of research ideas and equitable partnerships guided under the HCA Ethics Working Group is our priority. To enable the advancement of such large-scale projects, we will aim to implement ethically responsible, socially robust, and legally compliant research from the beginning. Continuous monitoring in the form of yearly consortium meetings should be tasked with the analysis of emerging ethical and societal aspects from the use of these new technologies. These meetings will also attempt to identify weaknesses or areas of inadequate progress and take actions to flag areas of expertise that are missing. The task forces formed during the initial meetings should meet regularly to apply concrete changes for patient groups, researchers, or data collectors.

## Conclusions

In sum, there is an enormous opportunity for integrated and precise oral health care initiatives that leverage the accessibility of this space to improve oral and systemic health. Profiling of these conditions from a patient-centered perspective will increase our understanding of disease cellular origin, mechanisms, etiology, and diagnostics—rendering them experimentally tractable to test new hypotheses for better diagnosis and drug discovery. To achieve this grand vision requires the collaboration of a multidisciplinary international team—spanning the basic and computational sciences to clinical practice (Fig. 4). This team science approach will be key to achieving inclusive, ancestrally diverse, open access, multiomic reference atlases of the human oral and craniofacial tissues and fluids across the life span. This open data resource will provide quantitative, multiscale information sufficient to build integrated prediction models of key oral and craniofacial cell and tissue states to develop breakthroughs in oral health for all. If you want to join the network, know more about our work, and collaborate with the network either by facilitating access to samples or by contributing with data sets for the OCBN atlas, please contact oral@humancellatlas.org.

## Author Contributions

A.J. Caetano, contributed to data analysis, drafted and critically revised the manuscript; I. Sequeira, K.M. Byrd, contributed to conception, design and data analysis, drafted and critically revised the manuscript; P.T. Sharpe, A. Kimple, M.A. Shazib, A. Opasawatchai, B.M. Warner, J. Krivanek, K. Kretzschmar, L.K. McKay, M. Freire, O. Klein, P.R. Tata, S. Pringle, S. Teichmann, D.W. Williams, I. Miller Zmora, Q. Easter, W.J. Lu, P. Perez, T. Pranzatelli, S. Lwin, R. Boucher, M. Bush, C.D. Conde, M. Haniffa, J.S. Hagood, A.A. Volponi, V. Yianni, J. Macken, M. Efremova, E. Todres, B. Matuck, F. Fortune, A.O. Pisco, D. Boffelli, Y. Kapila, F. Momen-Heravi, A. Gulati, N. Moutsopoulos, P. Beachy, S. Wallet, T. Biddinger, D. Pereira, R. Kumar, contributed to data analysis, critically revised the manuscript. All authors gave final approval and agree to be accountable for all aspects of the work.
